# Infection with SARS-CoV-2 causes flares in patients with juvenile idiopathic arthritis in remission or inactive disease on medication

**DOI:** 10.1186/s12969-021-00653-8

**Published:** 2021-11-29

**Authors:** Boris Hügle, Manuela Krumrey-Langkammerer, Johannes-Peter Haas

**Affiliations:** German Center for Pediatric and Adolescent Rheumatology, Gehfeldstrasse 24, 82467 Garmisch-Partenkirchen, Germany

## Abstract

**Background:**

Flares of juvenile idiopathic arthritis (JIA) have been described in the context of various infections. Flares of rheumatic diseases in adults have been described following infection with SARS-CoV-2 in several cohorts. So far, the effect of infection with SARS-CoV-2 on the course of JIA is unknown.

**Methods:**

The database of the German Center for Pediatric and Adolescent Rheumatology was searched for patients with confirmed infection with SARS-CoV-2 and subsequent disease flare, admitted from July 2020 until June 2021. cJADAS-27, ESR and C-reactive protein, as well as uveitis activity, medication at the time of flare and treatment of flare was extracted. Patient cases were described individually.

**Results:**

Out of 988 patients admitted, five patients with remission off medication (*n* = 2) or inactive disease on medication (*n* = 3) were identified, with flare symptoms up to four weeks after infection with SARS-CoV-2.

**Conclusions:**

Flares can occur after infection with SARS-CoV-2 in patients with JIA in remission or inactive disease on medication. Treating physicians need to be aware of this fact, especially when counseling patients with rheumatic diseases about the respective dangers of COVID-19 and vaccination against SARS-CoV-2.

## Background

Juvenile idiopathic arthritis (JIA) is the most common chronic rheumatic disease in children, with a reported prevalence that varies between 16 and 150 per 100,000 [1].

The course of JIA may have multiple periods of inactive disease and flares of inflammatory activity [2].

The COVID-19 pandemic, caused by SARS-CoV-2 infection, has seen at least 190 million confirmed cases and over 4 million deaths worldwide recorded until July 2021. In most people the infection will cause a mild-​to-​moderate flu-​like illness characterized by fever, cough, and loss of taste and smell, among other symptoms; some patients require hospitalization and ventilatory support and are at acute risk of dying [3]. In adults with rheumatic diseases, survey data and a retrospective study in a Latino cohort has shown an increased risk of flare after COVID-19 [4–6].

Children are among the least affected by the disease, with COVID-19 less frequent and less aggressive in this age group [7]. Rare cases of an inflammatory multisystem syndrome following infection with SARS-CoV-2 have been describes in children, however (PIMS-TS) [8]. About the risk of flare in JIA due to coronavirus infection, little is known.

We report five cases of patients with JIA in remission or long-term inactive disease on medication who developed flares of their disease in close temporal correlation with a confirmed prior diagnosis of SARS-CoV-2 infection.

## Methods

The database of the German Center for Pediatric and Adolescent Rheumatology in Garmisch-Partenkirchen was searched for patients with serologically confirmed coronavirus infection and subsequent flares of their rheumatic disease. A flare was defined as new signs of arthritis or uveitis, i.e. an increase in the total joint count or JADAS or increase in anterior chamber cells or flare requiring a modification in therapy, following a period of inactive disease.

Inclusion criteria were: 1) a diagnosis of juvenile idiopathic arthritis according to ILAR criteria [9], 2) flare of the disease in close temporal correlation, i.e. within three months, to a confirmed infection with SARS-CoV-2, and 3) no change to treatment after the last flare-up of the disease until the current flare including no pauses in medication documented in the patient file. Explicitly excluded were patients who reduced or stopped medication as a consequence of diagnosis of infection with SARS-CoV-2, and subsequently developed a flare of their rheumatic disease. The following data was extracted from the registry: cJADAS-27, ESR and C-reactive protein at the time of flare and at the last assessment prior to the flare, uveitis activity according to SUN criteria, medication at time of flare and treatment of the flare. As a matter of routine, all patients were required to show negative antigen testing (SARS-CoV-2 Rapid Antigen Test, Roche, Grenzach-Wyhlen, Germany) and PCR testing (Vivalytic SARS-CoV-2 PCR-Test, Bosch Healthcare, Waiblingen, Germany) prior to admission, and patients in whom a preceding infection was suspected received quantitative determination of SARS-CoV-2-IgG by ELISA.

## Results

In 988 cases with JIA admitted from July 2020 until June 2021 to the German Center for Pediatric and Adolescent Rheumatology, serology for COVID-19 was determined in 178 cases. Of these, 13 samples were positive. Six patients had no signs of arthritis flare but a recent history of other symptoms compatible with infection with SARS-CoV-2. Two had signs of arthritis flare after discontinuation of medication following the diagnosis of COVID-19. The remaining five patients with flare after infection with SARS-CoV-2, despite no change in medication, are presented here (Table 1). Laboratory data and treatment of the flare is given in Table 2. No patient showed any sign of uveitis at time of flare. Prior to admission, the patients had been seen by their local pediatric rheumatologists on a regular, usually three-monthly basis, where inactive disease was confirmed.

**Table 1:** Demographic and clinical data of the patients

Case number	Age	Diagnosis according to ILAR criteria	Age at Diagnosis (years)	Age at last visit (years)	Duration of Remission (months)	Clinical signs of SARS-CoV-2 infection	Affected joins	Estimated time between infection and arthritis flare	Medication
**1**	7	extended Oligoarthritis	2.8	7.6	40.8	none	right ankle	3 weeks	none
**2**	17	Rheumatoid-factor negative Polyarthritis	11.5	18.5	5.8	none	both ankles and subtalar joints	3 weeks	baricitinib
**3**	9	Seronegative Polyarthritis	0.7	8.9	12.2	fever, diarrhea	both knees, right ankle	1 week	methotrexate, etanercept
**4**	17	Enthesitis-associated arthritis	10.9	18.1	41.5	respiratory symptoms, loss of taste	right knee, ankles, wrists, fingers	4 weeks	none
**5**	15	extended Oligoarthritis	2.9	15.6	20.5	fever, respiratory symptoms	wrists, hips, knees, ankles	4 weeks	golimumab, etanercept

**Table 2:** Clinical and laboratory values, and treatment of flare of the patients

Case number	Age	CRP on last admission prior to flare (mg/dl)	CRP at time of flare (mg/dl)	ESR on last admission prior to flare (mm/h)	ESR at time of flare (mm/h)	cJADAS − 27 on last admission prior to flare	cJADAS-27 at time of flare	Treatment
**1**	7	0,09	0,83	7	11	1	3	IACSI, NSAID
**2**	17	0,02	0,2	2	18	N/A	7	methylprednisolone pulses × 3
**3**	9	< 0,06	0,11	10	10	1	15	methylprednisolone pulses × 3
**4**	17	0,35	18,11	3	73	6	20	methylprednisolone pulses × 3, IACSI, addition of methotrexate
**5**	15	0	0,22	5	31	1	10	methylprednisolone pulses × 3, IACSI, switch from golimumab to abatacept

### Patient cases

*Case 1* is a seven-year-old girl diagnosed with extended oligoarthritis at the age of two years and ten months with arthritis in knees, ankles and, in the course of the disease, the left subtalar joint and both temporomandibular joints. She had been treated with methotrexate and later with adalimumab. Medication was stopped approximately 18 months ago and she continued well without clinical signs of arthritis.

She presented, off all medications, with swelling of the right ankle. Approximately three weeks prior to admission, several cases of COVID-19 were diagnosed in the family but the patient had no clinical signs and repeatedly negative antigen tests. Ankle swelling started shortly thereafter. On admission, she tested highly positive for SARS-CoV-2-IgG (> 120.00 relative units/ml), but negative for IgM antibodies and PCR and antigen testing.

*Case 2* is a 17-year-old adolescent male who had been diagnosed with rheumatoid-factor negative polyarthritis at the age of 11 years and six months. He was treated with intraarticular steroid injections, methotrexate and in the course of his disease, etanercept, adalimumab and tocilizumab. He was switched to baricitinib 6 months prior to admission and experienced a complete response, with confirmed inactivity by the treating physician. However, he presented with arthritis in both ankle and subtalar joints three weeks after COVID-19, with inapparent clinical symptoms diagnosed by antigen testing after his brother had developed a symptomatic infection with SARS-CoV-2.

*Case 3* is a nine-year-old girl who had been diagnosed with rheumatoid-factor negative arthritis at the age of ten months. She was initially treated with methotrexate, intraarticular steroid injections, and etanercept was added at age three years. She had occasional mild flares, the last one at age seven years where dosage was adjusted.

She presented on etanercept (approx. 0.7 mg/kg) with arthritis in both knees and the left ankle, both ongoing for three months. This had developed shortly after COVID-19 of the whole family (confirmed by antigen testing at that time) where she had fever and mild diarrhea.

*Case 4* is an 18-year-old adolescent male who had been diagnosed with enthesitis associated arthritis at age 11 years. He was treated with methotrexate, hydroxychloroquine and low-dose prednisolone until medication was stopped at the age of 17 years. He continued well without medication until he developed an infection with SARS-CoV-2, with positive antigen test, with signs of infection in the upper respiratory tract, vertigo and loss of taste. He developed increasing joint pains shortly thereafter and presented three months later with confirmed arthritis in the right knee, both wrists, ankles and several finger joints.

*Case 5* is a 15-year-old adolescent female who had been diagnosed with extended oligoarthritis starting at age three. She was treated with multiple intraarticular steroid joint injections and methotrexate, but was started on etanercept at age nine due to ongoing arthritis. At age ten she developed uveitis of the left eye, when she was switched first to adalimumab, then infliximab, and then due to antibodies to both, to tocilizumab. Due to ongoing arthritis she was switched to golimumab at age 13 which, together with methotrexate, led to lasting remission. She presented approximately 18 months later, four weeks after COVID-19, confirmed by antigen test, with arthritis in both wrists, hips, knees, ankles and the right temporomandibular joint.

## Discussion

Here we describe five cases of patients with JIA, in clinical remission or inactive on medication, who developed a flare of their disease shortly after a documented infection with SARS-CoV-2. Time between infection and flare can only be roughly estimated, as patients were asymptomatic in two cases and arthritis was in most cases assessed by a trained rheumatologist at a later date. In general, the time span between infection and start of joint symptoms appears to be between one and four weeks. Patients were on various medications, or off medication completely, but all were inactive for at least six months prior to the viral infection with SARS-CoV-2.

Frequency and clinical symptoms of acute respiratory infections in patients with chronic disease have been poorly studied. Respiratory infections in children with cystic fibrosis was not more frequent, but associated with higher morbidity compared to healthy controls [10]. Infectious diseases have, however, long been identified as a possible source of flare in JIA, especially *M. pneumoniae* and *C. jejuni* [11]. Flares have been associated with acute respiratory infections and influenza-like illness, although the majority of infections do not cause flares [12].

Even without prior infections, flares are frequent occurrences in JIA patients in inactive disease, with 42.5% of patients developing a flare within one year of achieving inactive disease [2]. However, there are indications that flare rate in rheumatic disease is increased after SARS-CoV-2 infection: in adults with rheumatic disease, 19% of patients with rheumatic disease hospitalized for SARS-CoV-2 showed signs of flare of their underlying rheumatic disease [13]. A recent study from Italy describes an increased flare rate in a single center in patients with JIA, with the flare rate overall rising from 6.3 to 16.9% compared to the previous year [14]. In a survey conducted in the German registry, disease activity measured by numeric rating scale increased more than one point in five of 32 paediatric patients with rheumatic disease [15]. Little is known regarding flares in JIA after COVID-19 vaccination, even if other vaccinations have been shown not to be associated with an increased risk [16]. A preliminary study from Greece did not show any disease exacerbation in 21 adolescents with JIA on etanercept following vaccination [17]. No flares after vaccination were observed in this institution.

Patients infected with SARS-CoV-2 have shown to exhibit a variety of elevated cytokine levels, most prominently IL-6 [18, 19]. The efficacy of treatments targeting these cytokines in COVID-19 has been demonstrated [20]. It has also been shown that the dysregulation of the immune system can last for a long time after the infection with SARS-CoV-2, and can cause long-term damage in different organ systems [21–23]. The role of cytokines in the pathogenesis of JIA has been established, and more recent data points at various roles especially of IL-6 in the different subtypes of JIA [24, 25]. It is therefore conceivable that a change in cytokine patters induced by SARS-CoV-2 leads to a flare of disease in an already predisposed person.

An early survey from Italy in adults showed the benefit of continuing rheumatic medications to stabilize the disease and prevent flares [26]. The American College of Rheumatology Guidance for COVID-19 vaccination in patients with rheumatic diseases also recommends continuation of most medications in JIA after vaccination, with the exception of methotrexate and abatacept, where a pause of one week is warranted [27]. Given that additional patients were seen in this cohort with flare after discontinuation of medication following a diagnosis of COVID-19, it seems a sensible course of action to continue antirheumatic medications after an infection with SARS-CoV-2. In fact, it would be worthwhile to consider optimizing the dosage of conventional synthetic and biologic DMARDS after confirmed infection with SARS-CoV-2.

This is a retrospective case series, with no definitive proof of pathophysiological correlation between documented infection and observed flare. Further studies in a larger cohort are necessary to determine the risk of flare in the JIA population and possible ways to prevent this, beyond the obvious benefits of vaccination.

## Conclusions

In conclusion, we describe flares after infection with SARS-CoV-2 in patients with JIA in remission or inactive disease on medication. Paediatric rheumatologists need to be aware of this fact in their daily practice, and the possibility of flares despite adequate medication might add emphasis to the recommendation to vaccinate against COVID-19. However, more studies are necessary to establish the true risk of flares in JIA from COVID-19, including the risk of vaccination against SARS-CoV-2.

## Data Availability

The datasets used and/or analyzed during the current study are available from the corresponding author on reasonable request.

## Abbreviations

COVID: Coronavirus Disease
JIA: Juvenile Idiopathic Arthritis
ELISA: Enzyme-Linked Immunosorbent Assay
SARS-CoV-2: Severe Acute Respiratory Syndrome Coronavirus-2
IACSI: Intra-articular Corticosteroid Injection
NSAID: Non-steroidal Anti-inflammatory Drug
